# Emerging Concepts about NAIP/NLRC4 Inflammasomes

**DOI:** 10.3389/fimmu.2014.00309

**Published:** 2014-07-02

**Authors:** Silvia Lucena Lage, Carla Longo, Laura Migliari Branco, Thaís Boccia da Costa, Carina de Lima Buzzo, Karina Ramalho Bortoluci

**Affiliations:** ^1^Centro de Terapia Celular e Molecular (CTC-Mol), Universidade Federal de São Paulo, São Paulo, Brazil; ^2^Departamento de Ciências Biológicas, Universidade Federal de São Paulo, São Paulo, Brazil

**Keywords:** NAIP, NLRC4, flagellin, caspase-1, inflammasomes, lysosomes, cell death

## Abstract

Neuronal apoptosis inhibitory protein (NAIP)/NOD-like receptor (NLR) containing a caspase activating and recruitment domain (CARD) 4 (NLRC4) inflammasome complexes are activated in response to proteins from virulent bacteria that reach the cell cytosol. Specific NAIP proteins bind to the agonists and then physically associate with NLRC4 to form an inflammasome complex able to recruit and activate pro-caspase-1. NAIP5 and NAIP6 sense flagellin, component of flagella from motile bacteria, whereas NAIP1 and NAIP2 detect needle and rod components from bacterial type III secretion systems, respectively. Active caspase-1 mediates the maturation and secretion of the pro-inflammatory cytokines, IL-1β and IL-18, and is responsible for the induction of pyroptosis, a pro-inflammatory form of cell death. In addition to these well-known effector mechanisms, novel roles have been described for NAIP/NLRC4 inflammasomes, such as phagosomal maturation, activation of inducible nitric oxide synthase, regulation of autophagy, secretion of inflammatory mediators, antibody production, activation of T cells, among others. These effector mechanisms mediated by NAIP/NLRC4 inflammasomes have been extensively studied in the context of resistance of infections and the potential of their agonists has been exploited in therapeutic strategies to non-infectious pathologies, such as tumor protection. Thus, this review will discuss current knowledge about the activation of NAIP/NLRC4 inflammasomes and their effector mechanisms.

## Introduction

Inflammasomes are multiprotein platforms containing specialized cytosolic sensors for a wide range of pathogen-associated molecular patterns (PAMPs) or damage-associated molecular patterns (DAMPs) that are able to activate the inflammatory caspase-1 and caspase-11 (caspase-4 in humans) in a manner dependent or independent of adaptor molecules (1–4). Inflammasomes are composed of a cytosolic receptor from the nucleotide-binding domain-leucine-rich repeat (NBD-LRR) [also named NOD-like receptors (NLR)] or the pyrin and HIN domain-containing protein (PYHIN) families; the adaptor molecule ASC [apoptosis-associated speck-like protein containing a caspase activating and recruitment domain (CARD)]; and pro-caspase-1 or pro-caspase-11. AIM2 is the only member of the PYHIN family described to form inflammasomes. AIM2 is composed of two domains: a C-terminal HIN200 domain and an N-terminal pyrin (PYD) domain. The members of the NLR family contain three domains: a central NBD that is responsible for protein oligomerization and common to all members; a C-terminal region composed of LRR sequences that are supposed to sense PAMPs or DAMPs; and an N-terminal portion that is responsible for the specificity of their molecular interactions and, therefore, their effector functions. The NLR proteins can be classified into NLRBs [NLR containing the baculovirus inhibitory (BIR) domain], NLRCs (NLRs containing the CARD domain), and NLRPs (NLRs containing the PYD domain) (5).

NOD-like receptor proteins are maintained in an autoinhibited state under physiological conditions. After agonist recognition, they undergo a conformational rearrangement, triggering the NBD domains. Then, these proteins expose the effector domain to allow the assembly of oligomeric complexes. The NLRs that lack the CARD domain to recruit and activate pro-caspases-1 and 11 require the assistance of the adapter molecule ASC, which contains the PYD and CARD domains for binding caspases (6, 7). The NLRC members can directly recruit pro-caspase-1 through homotypical interactions between CARD domains, or they can recruit the adaptor ASC to activate caspase-1 (2). The canonical effector mechanisms mediated by caspase-1 are the maturation and secretion of IL-1β and IL-18 and the induction of pyroptosis, a pro-inflammatory form of cell death. Furthermore, caspase-11 seems to be able to induce pyroptosis (8).

After a decade of inflammasome discovery (9), little is known about the molecular complex formed by most members of the NLR family. AIM2, NLRP3, and NLRC4 are the best-characterized inflammasome complexes. The importance of these complexes to control bacterial, viral, fungal, and protozoan infections and their influence in inflammatory processes are gaining prominence in the literature, although their precise activation mechanisms remain to be elucidated. Here, we focus on NLRC4 inflammasomes, the recent advances in the understanding of their assembly and the consequences of their activation to the immune response.

## Assembly and Activation of NAIP/NLRC4 Inflammasomes

The first reports about the recognition of cytosolic flagellin, the monomeric subunit from flagella present in motile bacteria, demonstrated that the neuronal apoptosis inhibitory protein (NAIP)-5 was responsible for the detection of cytosolic flagellin from *L. pneumophila* and for the restriction of infection (10, 11). In the same year, studies with *S. typhimurium* revealed that another member of the NLR family, NLRC4, was also able to detect cytosolic flagellin (12, 13). NLRC4 was first described in 2001 as a mammalian protein homologous to CED4 of *C. elegans*, whose function is to recruit and activate caspases through its CARD domain (14, 15). Because of the ability to activate caspase-1, previously known as interleukin-1-converting enzyme (ICE), NLRC4 was first named IPAF (ICE-protease-activating factor). Although the involvement of NLRC4 in the control of infections was previously reported, their agonists remained a mystery until 2006.

Flagellin is one of the best-characterized agonists of the innate immune system. Extracellular flagellin is recognized by TLR5 (16) but it can be delivered to the cell cytosol though the secretion systems present in virulent bacteria strains, such as the *S. typhimurium* type III secretion system (T3SS SPI-1) and *L. pneumophila* type IV (T4SS). In the cell cytosol, flagellin induces the formation of the NAIP5/NLRC4 inflammasome, leading to the subsequent activation of caspase-1 (17, 18, 23). Notably, the activation NAIP5/NLRC4 inflammasomes by cytosolic flagellin occurs independently of TLR5 (20), and these two receptors recognize distinct regions of flagellin (16). TLR5 senses a region present in the D1 domain of the protein, whereas the amino acid sequences recognized by NAIP5/NLRC4 inflammasomes are in the D0 domain of the molecule (18, 23, 19, 21, 22).

Previous studies have pointed to the involvement of NAIP5 in controlling *L. pneumophila* flagellated bacteria (24, 25) and to the involvement of NLRC4 in caspase-1 activation and the induction of macrophage death (14, 15), although the role of flagellin in these processes was unidentified at that time. The simultaneous demonstration of cytosolic flagellin recognition by NAIP5 and NLRC4 prompted a model that proposed the existence of two distinct inflammasomes that recognize slight differences in the structure of flagellin (10–13). In 2008, with the advent of NAIP5-deficient mice, Lightfield and collaborators confirmed that NAIP5 is required for NLRC4-containing inflammasome activation in response to *L. pneumophila* infection in a flagellin-dependent manner; however, the NLRC4-mediated macrophage responses against *S. typhimurium* were only partially dependent on NAIP5 (21). A subsequent work from the same group demonstrated that the differential requirement for NAIP5 in response to *S. typhimurium* and *L. pneumophila* infection is not due to intrinsic differences between distinct flagellins, as a genetically engineered *L. pneumophila* developed to express the *S. typhimurium* flagellin also activated the NLRC4 inflammasome in a manner strictly dependent on NAIP5 (17). These data indicated that another agonist from *S. typhimurium* could activate NLRC4 independent of the presence of NAIP5. In fact, these studies confirm that NLRC4 responds to the *S. typhimurium* PrgJ protein independently of NAIP5, thus explaining why NLRC4-mediated responses to *S. typhimurium* are only partially dependent on NAIP5.

The inflammasome structure formed by these proteins was unveiled only recently when two independent groups proposed a model for NAIP5/NLRC4 inflammasome assembly (18, 23). Using the transfection of inflammasome components and microbial molecules in HEK 293T cells or followed by biochemical assays, the authors demonstrated the ability of flagellin from different bacterial species to bind NAIP5. This interaction was dependent on the three leucine residues of the C-terminal portion of flagellin, confirming prior data (17). Furthermore, after the recognition of flagellin, a physical association between NAIP5 and NLRC4 was demonstrated, resulting in the formation of an oligomeric complex. Reconstitution experiments using truncated receptor variants showed that NAIPs are upstream of NLRC4 and suggest that they interact via the NBD domain. Notably, NAIP6 worked similarly to NAIP5, as it induced the oligomerization of NLRC4 in response to flagellin, and this could explain the response of NAIP5^−/−^ cells to high concentrations of flagellin. NAIP1 and NAIP2 also recruit NLRC4 in response to the bacterial needle and inner rod proteins of T3SS, respectively (18, 23). Therefore, NAIP proteins seem to be the universal sensors of cytosolic flagellin and secretory complex proteins, whereas NLRC4 acts as an adapter molecule and is responsible for the recruitment and activation of caspase-1. It is noteworthy that there is only one functional NAIP found in humans, which is not activated by flagellin but is able to detect needle proteins of T3SS, similar to NAIP1 (18).

Despite these recent contributions to the understanding of NAIP/NLRC4 assembly, the molecular requirements of bacterial proteins for the formation of the inflammasome complex still requires further clarification. Lightfield et al. (21) originally demonstrated that the final 35 amino acids of the C-terminal portion of the flagellin molecule are essential for the activation of NAIP5. Moreover, the replacement of three leucine residues by alanine in this region abrogated the potential of flagellin to activate NAIP5. However, these studies were based on constructs containing only the C-terminal portion of the flagellin structure. A recent study using whole flagellin with or without these regions have shown that although the three leucine residues were essential for the detection of the C-terminus, their involvement seems to be less important for full-length flagellin recognition, as whole flagellin containing three alanines instead of three leucines still induces cell death and inflammasome complex formation, although fewer complexes are formed (22). Surprisingly, although the absence of the N-terminal domain does not affect the ability of whole flagellin to interact with NAIP5, constructs containing only N-terminus also retain the ability to activate NAIP5/NLRC4. Thus, the molecular interaction between flagellin and NAIP5/6 still requires clarification. Moreover, although flagellin was found inside the NAIP5/NLRC4 complex, as demonstrated by immunoprecipitation (19, 26) and yeast two-hybrid (18) assays, providing a basis for the model of direct interaction between flagellin and NAIP5, our group recently demonstrated the ability of cytosolic flagellin to activate a lysosomal pathway and the requirement of cathepsin B for NLRC4-dependent IL-1β secretion and pyroptosis (27). These observations raise the possibility that NAIP5/NLRC4 can also be activated by cytosolic alterations induced by the presence of flagellin, as proposed for the activation of the NLRP3 inflammasome (28).

Challenging prior models that hypothesized that LRR domains are responsible for the detection of NLR agonists, a recent study found that these domains are dispensable for the ligand specificity of NAIPs (26). By using a series of chimeric proteins in which the N-terminal domains of NAIP5 or NAIP6 were fused to the C-terminal domains of NAIP2 or vice-versa, the authors demonstrated that NAIP proteins lost the ability to oligomerize with NLRC4 only when NOD domain-associated α-helical domains were absent, suggesting that ligand specificity maps to this region. Interestingly, a similar region in NLRC4 was recently associated with its autoinhibition (29), whereas LRR domain from NAIPs was shown to be required for the maintenance of this protein in an autoinhibited conformation (19). Despite, these unsolved pieces of the puzzle, it has been demonstrated that the interaction of NAIPs with their ligands and the association of NLRC4 with NAIPs induce conformational changes in these molecules that enable their oligomerization and activation (22, 30). Predicted models for the NAIP/NLRC4 inflammasome suggest that these complexes contain an excess of NLRC4 for each NAIP protein (22, 26) and that NLRC4 molecules are able to recruit and activate caspase-1 either directly or through an ASC adapter. The association of pro-caspase-1 with an inflammasomes-containing ASC allows its autoproteolytic cleavage to become an enzymatically active heterodimer capable of processing pro-IL-1β and pro-IL-18 into mature cytokines (2). In contrast, an ASC-independent complex activates caspase-1 without autoproteolysis, which is sufficient for caspase-1 to target a distinct subset of substrates critical for the induction of pyroptosis.

## Canonical Effector Mechanisms Induced by NAIP/NLRC4 Inflammasomes

### Pyroptosis

The NAIP5/NLRC4 inflammasome is perhaps the best-studied inflammasome complex with regard to resistance to infections. Their involvement has been reported against infections such as *S. typhimurium* (31, 32), *L. pneumophila* (25), *P. aeruginosa* (33, 34), *Y. pestis* (35), *S. flexneri* (36), and *A. veronii* (37). NAIP/NLRC4-mediated responses are related to the restriction of bacterial growth due to the active caspase-1-mediated canonical and non-canonical effector mechanisms, highlighting the importance of this inflammasome as a host defense mechanism against a large number of bacterial infections. The best elucidated effector mechanisms involved in the control of infections mediated by caspase-1 are the secretion of inflammatory cytokines IL-1β and IL-18 and the induction of pyroptosis (38).

The term pyroptosis (from the Greek “pyro” meaning fire or fever, and “ptosis” to a fault) was coined in 2001 to describe a pro-inflammatory programed cell death during *S. typhimurium* infection (39). Morphological and biochemical changes displayed by *S. typhimurium*-infected dying cells were more closely related to those found in classic necrosis compared with those observed during apoptosis, including the following: (1) diffuse DNA fragmentation with no chromatin condensation; (2) early loss of membrane integrity observed by the simultaneous uptake of annexin V with an impermeable membrane dye; (3) lactate dehydrogenase (LDH) release, suggesting a loss of intracellular content; and (4) independence of any apoptotic caspase. Although cells dying by pyroptosis displayed features of necrosis with an inflammatory outcome, the authors found that this process was highly regulated by active caspase-1, as the addition of inhibitors of caspase-1 (z-YVAD-fmk) abolished *S. typhimurium*-induced cell death.

The induction of pyroptosis by pathogenic bacteria depends on an active secretion system that translocates bacterial proteins into the cell cytosol, such as the T3SS (SPI-1) of *S. typhimurium* and type IV (T4SS) of *L. pneumophila* (12, 13, 40–42). Mutant *L. pneumophila* (43) or *P. aeruginosa* (34, 44) lacking flagellin fail to activate caspase-1 and, therefore, are not able to induce pyroptosis and IL-1β secretion in infected macrophages. Accordingly, the transfection of purified flagellin from *L. pneumophila* and *S. typhimurium* directly into the cell cytosol is sufficient to trigger caspase-1-dependent pore formation, pyroptosis, and IL-1β secretion (45, 46). Importantly, infection with the non-flagellated bacteria *S. flexneri* also induces NLRC4-mediated pyroptosis, most likely in response to the inner rod component of T3SS (36).

Although the molecular mechanisms that regulate pyroptosis remain to be elucidated, the model of *S. typhimurium* infection has given us important knowledge about this form of cell death. The cell lysis observed during pyroptosis seems to result from a highly regulated process of pore formation in the plasma membrane (45, 46). Pores dissipate cellular ionic gradients but allow the retention of larger cytoplasmic constituents, leading to increased liquid osmotic pressure and water influx. These events are followed by cell swelling and subsequent osmotic lysis with the release of intracellular contents, which are potentially inflammatory (45, 46). Caspase-1-dependent DNA cleavage also occurs during pyroptosis (45, 47). However, the DNA cleavage observed during *S. typhimurium*-induced pyroptosis is independent of caspase-activated DNase (CAD) (45, 47), unlike what is observed during apoptosis, in which the proteolysis of inhibitor of CAD (ICAD) by apoptotic caspases mediates the release of CAD to the nucleus, where it cleaves DNA between nucleosomes. Therefore, pyroptotic cells do not display the typical pattern of oligonucleosomal fragmentation observed during apoptosis, a fact that can be used to distinguish between these two processes of cell death (48).

There is good evidence implicating pyroptosis as an important host defense mechanism mediated by NAIP/NLRC4 that clears intracellular pathogens *in vitro*. The death of infected macrophages by pyroptosis seems to correlate with a rapid loss of the replicative niche and high bacterial loads are recovered from macrophages deficient in components of inflammasomes or infected with mutant bacterial strains that fail to trigger their activation [reviewed by Bortoluci and Medzhitov (1) and Bergsbaken et al. (49)]. Moreover, a study conducted *in vivo* demonstrated that the NLRC4-dependent flagellin-mediated lysis of bacteria-containing macrophages not only results in the early loss of the intracellular replication niche but also creates an inflammatory milieu with the recruitment of effector cells to the infection site, which are involved in pathogen clearance (32). Although the possible targets of caspase-1 and caspase-11 mobilized during pyroptosis remain unidentified, the studies involving NAIP/NLRC4 hugely contribute to the idea that this inflammatory form of cell death is an important effector mechanism against infections.

### IL-1β and IL-18 secretion

IL-1 was the first identified cytokine and has been related to several inflammatory processes. IL-1 plays a role in virtually all cells and organs, ranging from fever and resistance to microorganisms to the activation of the hypothalamus–pituitary–adrenal axis (HPA) (50–56). IL-18 was first described in 1989 as a potent IFN-γ-inducing factor and an important component of polarized type-1 T helper cells (Th1) and type-1 macrophages (M1) responses, cells with a pro-inflammatory profile (57–59). Macrophages, monocytes, lymphocytes, keratinocytes, microglia, neutrophils, dendritic cells, and other cells are described as important sources of IL-1β and IL-18 (60–64). IL-18 and IL-1β have similar processing; they are both synthesized in an inactive form that requires processing by active caspase-1 to become biologically active (61, 65, 66). Although extensively studied, the mechanism responsible for IL-1β and IL-18 release has not been fully elucidated. These cytokines can be passively released during cell lysis; however, there is recent evidence supporting the existence of active mechanisms involved in the secretion of IL-1β and IL-18, such as caspase-1-induced membrane pores, vesicle shedding and lysosomal exocytosis (45, 49).

Although the precise effector mechanisms of IL-1β and IL-18 remain to be elucidated, these cytokines have been reported to be important mediators induced by NAIP/NLRC4 to host resistance to bacterial infections (67). In addition to the effects of IL-1β and IL-18 in the activation and recruitment of innate immune cells, these cytokines have important roles in the activation and differentiation of T lymphocytes (52). IL-1β and IL-18 have been shown to drive the establishment of T CD4^+^ adaptive responses in mice and in humans and are responsible for the differentiation of Th17 and Th1, respectively (68–70). However, little is known about the involvement of IL-1β and IL-18 in NAIP/NLRC4-induced adaptive immune responses. Kupz et al. demonstrated that IL-18, when produced by the activation of NLRC4 during infection by *S. typhimurium*, is required for the activation of non-cognate CD8^+^ T cells and the production of IFN-γ (71), supporting a role for this cytokine in the induction of cellular responses.

Additional evidence of the role of NAIP/NLRC4 in the activation of T cells came from an experimental vaccination with irradiated flagellin-expressing tumor cells. Authors demonstrated that the immunization of mice with flagellin-fused tumor cells induced tumor-specific CD4^+^ and CD8^+^ T cell responses and prevented parental tumor growth. Despite the well-known role of TLR5, the recognition of flagellin by the NAIP5/NLRC4 inflammasome was also required for the induction of a protective CD8^+^ T cell response and tumor suppression. Although the NAIP5/NLRC4 inflammasome-mediated IL-1β secretion in response to the injection of flagellin-modified tumor cells, it is unclear whether the involvement of this cytokine was necessary for the success of this immunotherapy. The role of IL-1β and IL-18 in tumorigenesis remains controversial. There is strong evidence supporting pro-tumorigenic properties of these cytokines via the induction of chronic inflammation. Although the induction of Tregs and Th17 could impair the immune response against tumor cells, it is reasonable to consider that the activation of Th1 and cytotoxic CD8 T cells by IL-1β and IL-18 may be beneficial to the host (72, 73).

## Emerging Effector Mechanisms Mediated by the NAIP/NLRC4 Inflammasome

### Humoral effector mechanisms

In addition to the well-characterized functions of NAIP/NLRC4 inflammasomes described above, non-canonical effector mechanisms have emerged. Recent data describe a range of effector functions mediated by NAIP/NLRC4 inflammasomes that operate independently of IL-1β, IL-18 and pyroptosis. The NAIP5/NLRC4 inflammasome has been implicated in the activation of phospholipase A2 (cPLA2) with a consequent production of lipid mediators, such as prostaglandins and leukotrienes (74). Authors demonstrated that systemic cytosolic flagellin stimulation leads to an “eicosanoid storm” that initiates inflammation and the loss of vascular fluids, resulting in a very fast death in mice. Of note, these effects are mediated by NAIP5/NLRC4 and occur independently of IL-1β/IL-18 or pyroptosis.

Inflammasomes have also been implicated in the active secretion of endogenous molecules known as DAMPs, challenging the idea that these molecules are only passively released during the process of cell lysis (75). IL-1α is an alarmin, whose release has been recently linked to inflammasomes. Both IL-1β and IL-1α present some common features, such as belonging to the same family, synthesis in the cytoplasm and secretion by an unconventional pathway independent of the endoplasmic reticulum and Golgi complex (55); additionally, they are released simultaneously by various stimuli, and they act on the same receptor, IL-1R1, thus sharing some biological functions (52). However, despite these similarities, there are some important differences in the production, secretion, and function of these cytokines. Unprocessed forms of both IL-1α and IL-1β are thought to be produced in response to TLR ligands, but they have distinct activities. Unlike IL-1β, which needs to be processed by caspase-1 to become biologically active (65), the uncleaved form of IL-1α is able to engage IL-1R1 (60, 76), although it’s full activity seems to require cleavage by calpain (77). Although IL-1α is not a substrate for caspase-1, there are some reports that have demonstrated that macrophages from caspase-1-deficient mice release less IL-1α (27, 78–80), suggesting the involvement of inflammasomes.

The mechanism by which caspase-1 mediates IL-1α secretion is still a matter of debate. A recent study demonstrated that the requirement of inflammasomes for IL-1α secretion depends on the nature of agonists (81). Caspase-1 has been described as a shuttle that facilitates the secretion of leaderless proteins, such as IL-1α (80). However, it is not clear whether active caspase-1 is the shuttle itself or whether it activates another enginery that is dependent on its activity, e.g., IL-1β (82) or IL-1R2 (77), as has been proposed for the secretion of IL-1α in response to NLRP3 agonists. The involvement of NLRC4 inflammasomes in IL-1α secretion is poorly understood. In one previous study, infection by *S. typhimurium* resulted in NLRC4- and caspase-1-dependent secretion of IL-1α (81). Interestingly, in contrast with most of the NLRP3 agonists, the secretion of IL-1α in response to *S. typhimurium* was completely independent of ASC, indicating a differential requirement for this adaptor molecule in cytokine secretion in response to NLRC4 agonists, as IL-1β is entirely dependent on ASC (2). However, Barry et al. showed that IL-1α initiates the inflammatory response driven by *L. pneumophila* independent of caspase-1 and NLRC4 (83). We recently reported that the activation of macrophages with purified flagellin inserted into lipidic vesicles induced IL-1α secretion in a manner partially dependent on caspase-1 and cathepsin B (27). Therefore, the reasons for the discrepancies in the literature and the precise mechanisms involved in the cross talk between IL-1α and NLRC4/caspase-1 axis remain to be addressed.

Another factor whose secretion has been linked to inflammasomes is the “High Mobility group box-1” (HMGB-1). HMGB-1 is a nuclear protein involved in the regulation of nucleosome function and DNA transcription that functions as an inflammatory mediator when released to the extracellular milieu (84). Lamkanfi et al. reported a critical role for HMGB-1 secreted through the NLRP3/ASC/caspase-1 axis in LPS-induced endotoxic shock (85). Interestingly, macrophages infected with *S. typhimurium* released significant amounts of HMGB-1 in a NLRC4 and caspase-1-dependent manner but independently of ASC, which is similar to previous reports of IL-1α secretion (81). During pyroptosis induced by a variety of stimuli, including *S. typhimurium* infection, HMGB-1 did not undergo caspase-1-mediated processing before its secretion, but extracellular HMGB-1 was hyperacetylated at the nuclear localization sequences (NLSs) (86). Because this translational modification is essential for HMGB-1 translocation from the nucleus to the cytoplasm (87, 88), HMGB-1 release upon inflammasome activation seems to be a coordinated process. More recently, Nystrom et al. (89) reported that NLRC4-mediated pyroptosis is the prevalent factor in the regulation of HMGB-1 secretion, leading to the release of the chemotactic acetylated HMGB-1 isoform without requiring TLR-derived priming. Although the mechanisms by which inflammasome components can regulate DAMPs secretion still need to be better understood, DAMPs are already considered important therapeutic targets because of their role in host resistance against infection and their involvement in inflammatory disorders.

With respect to antibodies production NLRC4, NAIP5, and caspase-1 have been reported to have a redundant role with TLR5 in the induction of total IgG (90) or IgG1 (91) against flagellin or co-administered OVA and an additive effect to TLR5 in the induction of IgG2a (91). In the absence of MyD88, in which TLR5, IL-1β, IL-1α, and IL-18 signaling is compromised, the production of antibodies induced by flagellin was reduced but not abolished, and a large amount of antibodies was still produced (91). The same results were obtained with TLR5/caspase-1 double-knockout mice (91), supporting previous data that demonstrated that no significant difference was observed in specific anti-flagellin IgG titers in mice deficient for IL-18 (92) or IL-1R (93). These reports suggest that some yet-undiscovered mechanism that acts in addition to TLR5 and inflammasome-mediated cytokines could be involved in the adjuvant properties of flagellin, requiring new investigations into this agonist.

### Cellular effector mechanisms

In addition to inflammatory mediators and cell death processes, some cellular effector mechanisms mediated by NLRC4 have emerged. Previous studies from our group described a requirement of NAIP5, NLRC4, and caspase-1 for the activation of inducible nitric oxide synthase (iNOS) and nitric oxide (NO) secretion in response to cytosolic flagellin (94). Interestingly, cytosolic flagellin-induced iNOS activation is preserved in the absence of MYD88, ruling out the participation of TLR5, IL-1β, and IL-18. Moreover, NO secretion through the NAIP5/NLRC4-caspase-1 axis in response to flagellin is involved in the control of *L. pneumophila* (94) and *S. typhimurium* (unpublished data from our group) by macrophages, pointing to this pathway as an additional effector mechanism mediated by NAIP5/NLRC4.

Autophagy is another effector mechanism used by NAIP5/NLRC4 to control *L. pneumophila*. In the presence of NAIP5, NLRC4 macrophages present a rapid turnover of LC3^+^ *L. pneumophila*-containing vesicles, preventing the establishment of secondary infections (95). This response is mediated by the detection of flagellin, and the inhibition of autophagy in macrophages infected with flagellin-sufficient *L. pneumophila* increased the rate of pyroptosis in these cells. These data confirm a previous study that demonstrated that NLRC4 plays a role in the regulation of autophagy by binding Beclin-1 in steady-state conditions (96). Because the initiation of autophagy seems to precede the induction of pyroptosis, autophagy can be considered a pathway through which macrophages raise the threshold of contaminants necessary to result in the loss of cell by inflammatory cell death. NAIP5/NLRC4 can also restrict flagellin-competent *L. pneumophila* replication by promoting the delivery of *L. pneumophila*-containing phagosomes (LCP) to lysosomes for degradation (43, 97). In the absence of NAIP5/NLRC4/caspase-1, LCP avoids fusion with lysosomes, which allows the pathogen to exponentially replicate inside macrophages. This effect is dependent on caspase-1-mediated caspase-7 processing and does not require IL-1β/IL-18 and the classical apoptosis pathway involving caspase-8 and -9 (98). These data corroborate a previous report that demonstrated a requirement of NLRC4, caspase-1, and ASC for caspase-7 processing during infection with flagellin-competent *S. typhimurium* (99). NLRC4 and ASC-dependent caspase-8 proteolysis was also reported during *S. typhimurium* infection (100). Interestingly, caspase-8 contributes to *Salmonella*-induced IL-1β production, but it is dispensable for inducing pyroptosis, whereas caspase-1 processes pro-IL-1β and coordinates pyroptosis. These data highlight the fact that inflammasomes are dynamic complexes that are able to recruit distinct members of the caspase family to induce diverse effector functions in response to *Salmonella* infection.

Similar to what has been demonstrated during apoptosis (101, 102) and necrosis (103), the cleavage of PARP1 (also called ARTD1) was also observed during pyroptosis induced by *S. typhimurium* (104). PARP1 processing in *S. typhimurium*-infected macrophages was abrogated in *Nlrc4*^−/−^ but not in *Nlrp3*^−/−^ cells, consistent with the role of the NAIP5/NLRC4 inflammasome in the induction of pyroptosis during *S. typhimurium* infection (12, 31, 105). PARP1 is a nuclear chromatin-associated multifunctional enzyme that catalyzes the polymerization of ADP-ribose units from donor NAD^+^ molecules (106, 107). Although it has been historically studied in the context of genotoxic stress signaling and consequent apoptosis, PARP1 has been related to chromatin structure regulation, transcription, and chromosomal organization (108, 109). Previous reports showed that inflammasomes are able to use PARP1 to induce the transcription of NF-κB-dependent target genes independently of any type of programed cell death (110). Upon LPS stimulation, caspase-7 is activated by caspase-1, which is translocated to the nucleus to induce PARP1 cleavage at the promoters of a subset of NF-κB-dependent target genes that are negatively regulated by PARP1. Mutating the PARP1 cleavage site D214 renders PARP1 uncleavable and inhibits PARP1 release from chromatin and, therefore, chromatin decondensation, thereby restraining the expression of cleavage-dependent NF-κB target genes, such as *il-6, cfs2*, and *lif*, but not *ip-10* (110). Preliminary and unpublished data from our group suggest the involvement of caspase-1-dependent PARP1 cleavage in iNOS gene expression upon cytosolic flagellin stimulation, as iNOS expression is significantly reduced in macrophages that harbor non-cleavable PARP1 (D214N). This is important evidence of the involvement of inflammasomes in epigenetic regulation and gene expression, although many of these outputs require further evaluation.

**Figure 1:**
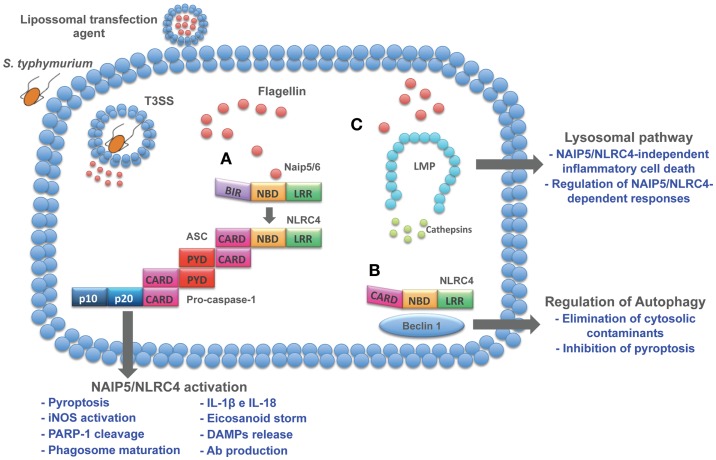
**Cytosolic pathways induced by flagellin**. Flagellin delivered to cell cytosol through bacterial secretion systems or transfection agents activates different pathways. **(A)** NAIP5/6-NLRC4 activation induces a series of cellular and humoral responses involved in host control of infections. **(B)** In resting cells, NLRC4 is complexed with Beclin-1, thus inhibiting autophagy. When flagellin is detected by NAIP5/6, NLRC4 is recruited to assembly inflammasome complex and release Beclin-1 to initiate autophagy. As a host protection response, autophagy is able to eliminate cytosolic cargo and inhibits pyroptosis, thus preventing cell loss and inflammation. Therefore, these emerging effector responses induced by flagellin open up new avenues to explore its immune potential as therapeutic targets. **(C)** Lysosomal destabilization leads to cathepsins release to cell cytosol, resulting in the induction of inflammasome-independent cell death that contributes to macrophage control of infection and regulation of NAIP5/NLRC4-dependent responses.

An important process of lysosomal exocytosis occurs during pyroptosis. Bergsbaken and Cookson (111) demonstrated that caspase-1-mediated pore formation induced during *S. typhimurium* infection promotes an influx of extracellular calcium, which is critical for lysosomal exocytosis. The release of lysosomal proteases with known antimicrobial activity contributes to the control of extracellular bacteria. In addition to the effect of lysosomal contents in the extracellular compartment, recent data from our group demonstrated that cytosolic flagellin is also able to activate a lysosomal pathway that culminates in an inflammasome-independent inflammatory form of cell death. This inflammasome-independent cell death induced by cytosolic flagellin is regulated by cathepsins B and D and is temporally correlated with the restriction of *S. typhimurium* infection by macrophages (27). Together, these data indicate a cross talk between lysosomes and NAIP/NLRC4 inflammasomes that impact the control of bacterial infections and opens new avenues for the development of inflammasome-based therapeutic strategies for non-infectious pathologies such as tumors. In fact, lysosomes have been considered important targets for the development of anti-tumor drugs (112). Lysosomes from cancer cells appear to be less stable than normal cells, which has given rise to the development of therapies based on lysosomotropic detergents. In this sense, flagellin could be an alternative that in addition to the induction of lysosomal cell death, is able to mediate several effector mechanisms as described throughout this review (Figure 1).

## Conclusion and Future Directions

More than 10 years after their discovery (14, 15), the molecular mechanisms involved in the activation of NAIP/NLRC4 began to be elucidated (18, 19, 26). From two distinct inflammasome complexes, NAIPs emerged as universal sensors for cytosolic bacterial proteins, whereas NLRC4 became an adaptor molecule responsible for the recruitment and activation of caspase-1. At the same time, in addition to NAIP5, novel NAIPs members were described, amplifying the potential of these proteins to detect bacterial infections (18, 19, 113, 114). Despite this important information, the molecular signatures of agonists recognized by NAIP/NLRC4 inflammasomes still require further study. Moreover, NLRC4 has been associated with host resistance against a mucosal *Candida albicans* infection (115) and in a colitis-associated colorectal cancer (CAC) model (116, 117). Interestingly, in both cases, NLRC4 seems to exert a protective role in non-hematopoietic compartments. However, the precise mechanism of NLRC4 activation in these models is unknown, raising the possibility that NLRC4 functions as an adaptor molecule for other NLR members in addition to NAIP and providing new insights into inflammasome signaling.

NAIP/NLRC4 are most likely the best-studied inflammasomes in the context of host resistance against infections. In addition to the extensively described IL-1β and IL-18 secretion and pyroptosis, other important effector mechanisms mediated by these inflammasomes have recently emerged (Figure 1). Moreover, flagellin, the best studied NAIP/NLRC4 ligand, has been reported to activate distinct pathways, such as autophagy (95) and a lysosome pathway (27) (Figure 1). Although the precise mechanism involved in the lysosome disruption by flagellin is still under investigation, it culminates in an inflammatory process of cell death that is accompanied by IL-1α secretion and contributes to the control of *S. typhimurium* by macrophages. This peculiar process of cell death occurs in the absence of inflammasome components. Additionally, the inhibition of cathepsin B disrupted IL-1β secretion and pyroptosis in response to cytosolic flagellin, indicating a role for lysosomal proteases in the regulation of NAIP/NLRC4-dependent responses. Because human cells do not express NAIP5 or NAIP6 (18), the activation of the lysosomal pathway by flagellin might be an alternative pathway used when human cells interact with flagellated bacteria that reach cell cytosol. In the context of therapeutic strategies, this knowledge could be an important gain, as the immune properties of flagellin have been extensively exploited in different models. At least in the context of anti-tumor vaccination (118) and antibody production (90, 91), the protective and adjuvancy roles of flagellin require its cytosolic detection. Together, these reports open up new avenues to explore the immune potential of NAIP/NLRC4 agonists as therapeutic targets.

## Conflict of Interest Statement

The authors declare that the research was conducted in the absence of any commercial or financial relationships that could be construed as a potential conflict of interest.
